# Patient participation and its determinants based on the Social Ecological Model: a cross-sectional study in North China

**DOI:** 10.1186/s12913-025-13795-2

**Published:** 2025-11-21

**Authors:** Xingyu Chen, Liping Cui, Lihua Wu, Dinuo Xin, Qian Zhang, Jing Ma, Ningning Li, Xiaohong Zhang, Wanling Li

**Affiliations:** 1 Department of Lymphoma, Tongji Shanxi Hospital, Shanxi Bethune Hospital, Shanxi Academy of Medical Sciences, Third Hospital of Shanxi Medical University, Taiyuan, 030032 China; 2 Department of Nursing, Shanxi Bethune Hospital, Shanxi Academy of Medical Sciences, Third Hospital of Shanxi Medical University, Tongji Shanxi Hospital, Taiyuan, 030032 China; 3 Department of Geriatrics, Tongji Hospital, Tongji Medical College, Huazhong University of Science and Technology, Wuhan, 430030 China

**Keywords:** Patient participation, Social Ecological Model, Determinants, Hierarchical regression, Shapley value decomposition

## Abstract

**Background:**

Grounded in the Social Ecological Model, this study aimed to assess the current status of patient participation in healthcare, identify its associated factors, and quantify the relative contribution of each factor to inform targeted interventions.

**Methods:**

A cross-sectional survey was conducted from May to October 2024 using convenience sampling at five tertiary hospitals in North China. A total of 573 patients were recruited. Data were collected through structured questionnaires, including the General Information Questionnaire, the Patient Participation Scale, the Patient Participation Competence Scale, the Patient Participation Attitude Scale, the Facilitation of Patient Involvement Scale, the Family APGAR Index, and the MacArthur Scale of Subjective Social Status.

**Results:**

The mean patient participation score was 3.56 ± 0.57, indicating a moderate overall level. Multilevel factors across the microsystem, mesosystem, and macrosystem influenced participation, with the microsystem exerting the strongest impact. Participation competence and attitude were the most influential determinants, followed by educational level, facilitation of participation, and family support. Additional factors included patients’ disease knowledge, socioeconomic status, residence, self-rated health, financial burden, age, and communication with healthcare providers.

**Conclusions:**

Patient participation in healthcare remains moderate, shaped predominantly by competence and attitudes. These findings highlight the need for healthcare policies and practices that empower patients with the skills, confidence, and supportive environments required for active engagement. Strengthening competence and attitudes, while addressing disparities related to education, socioeconomic status, and health literacy, may foster more equitable and participatory models of care.

**Supplementary Information:**

The online version contains supplementary material available at 10.1186/s12913-025-13795-2.

## Background

With the ongoing reform of China’s healthcare system, the patient-centered care model has increasingly replaced the traditional disease-centered approach. As a key component and practical manifestation of patient-centered care, patient participation refers to patients’ active engagement throughout their healthcare journey, including making informed decisions and utilizing available resources to manage their health conditions effectively [1]. Recognized as an important cornerstone of high-quality healthcare services [2], patient participation has been shown to not only improve treatment adherence, optimize clinical outcomes, and enhance patient satisfaction [3–5], but also contributes to better care quality, fewer medical errors, and improved patient safety [6–11]. In light of the global trend of population aging and the growing prevalence of chronic diseases, patient participation is expected to play an increasingly critical role in future disease management [12]. Conversely, insufficient participation can lead to poorer clinical outcomes, higher rates of hospital readmission, and escalating healthcare costs [9, 13, 14]. Recent advancements in mobile health technologies have significantly broadened access to health-related information. Along with improvements in public health literacy and legal awareness, these developments have empowered patients and increased their willingness to participate in healthcare [15]. While many studies indicate that patients hold positive attitudes toward participating in their own treatment and care [16–18], the actual level of participation remains significantly lower than their expressed willingness and needs [19–21]. Identifying the key determinants of patient participation is therefore essential to inform the development of evidence-based interventions that can effectively enhance patient participation in clinical care.

To date, studies have explored various factors influencing patient participation in healthcare, such as patients’ disease-related knowledge, participation attitudes and willingness, healthcare providers’ attitudes, and the quality of patient–provider communication [14, 22–24]. However, existing research presents several limitations. Most studies have focused on specific aspects of patient participation, such as shared decision-making or patient safety, while lacking a systematic examination of the entire participation process. Moreover, they have predominantly emphasized individual-level determinants, often neglecting multi-level contextual influences and their interactions. This has limited the development of a comprehensive framework that situates patients within their broader ecological environment and impedes the identification of the complex causes underlying low levels of participation. In addition, few studies have quantitatively assessed the relative contributions of influencing factors, thereby restricting the precision and effectiveness of targeted interventions. A systematic theoretical perspective is therefore needed to integrate multi-level determinants, comprehensively examine their effects on patient participation, and quantify the relative contribution of each factor.

The Social Ecological Model provides a critical framework for understanding the integrated effects of multi-level factors on individual behavior. It emphasizes that behavior is shaped and evolves through the interaction of the microsystem, mesosystem, and macrosystem [25]. Specifically, the microsystem refers to the individual level, including biological characteristics and psychological states; the mesosystem refers to small-scale social groups that directly interact with the individual, such as family and workplace; and the macrosystem refers to broader social environments such as communities, organizations, and sociocultural contexts [26]. The Social Ecological Model has been widely applied in studies examining the determinants of health-related behaviors [27, 28]. For instance, a study based on this model explored factors associated with health-promoting lifestyles among older adults and found that exercise frequency, smoking status, self-efficacy, health management, and frailty in the microsystem, family care in the mesosystem, and pre-retirement occupation, living area, access to community-based chronic disease services, and social support in the macrosystem significantly influenced health-promoting behaviors [29]. In addition, Crossman et al. [30] conducted a systematic review using this framework and identified multiple facilitators and barriers to adult sport participation across all three levels of the model. However, few studies have systematically analyzed the determinants of patient participation in healthcare from a multi-dimensional perspective grounded in the Social Ecological Model, incorporating microsystem, mesosystem, and macrosystem factors.

**Fig. 1:**
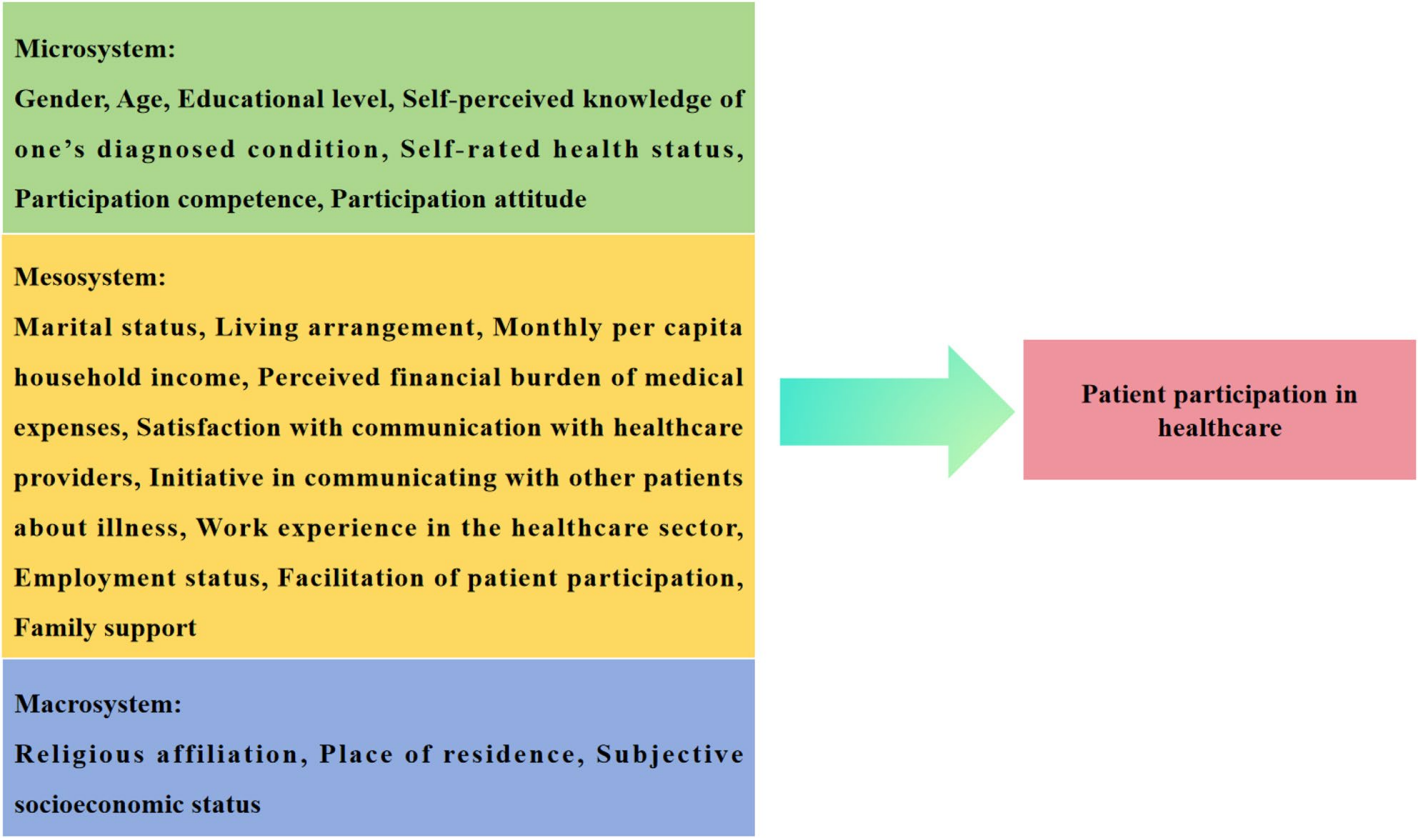
Theoretical model of factors influencing patient participation

Building on these insights, this study—guided by the Social Ecological Model—conceptualizes patient participation in healthcare as a behavior shaped by multi-level influences. It systematically examines the determinants of patient participation across the microsystem, mesosystem, and macrosystem, with the aim of providing a theoretical foundation for developing comprehensive and targeted intervention strategies. Based on an extensive review of existing literature, variables were categorized accordingly: the microsystem includes gender, age, educational level, self-perceived knowledge of one’s diagnosed condition, self-rated health status, participation competence, and participation attitude; the mesosystem includes marital status, living arrangement, monthly per capita household income, perceived financial burden of medical expenses, satisfaction with communication with healthcare providers, initiative in communicating with other patients about illness, work experience in the healthcare sector, employment status, facilitation of patient participation, and family support; and the macrosystem includes religious affiliation, place of residence, and subjective socioeconomic status. The hypothesized model of this study is presented in Fig. 1.

## Methods

### Ethical approval

This study was approved by the Medical Ethics Committee of Shanxi Bethune Hospital (Approval No. YXLL-2023-230). Written informed consent was obtained from all participants. The survey was conducted anonymously and voluntarily, and all responses were kept strictly confidential.

### Study design and participants

A cross-sectional study was conducted from May to October 2024. Participants were recruited using a convenience sampling method from five tertiary hospitals in Shanxi, China. Inclusion criteria were as follows: (1) aged 18 years or older; and (2) currently receiving treatment in either an outpatient clinic or an inpatient ward. Patients were excluded if they had a history of neuropsychiatric disorders that significantly impaired cognitive functioning.

Based on Kendall’s principle of sample size estimation, the minimum sample size should be 10 to 20 times the number of independent variables. Given that this study included 20 independent variables, the estimated sample size ranged from 200 to 400 participants. Considering an anticipated non-response or invalid response rate of 20%, the final calculated sample size requirement ranged from 263 to 525 participants.

### Questionnaire

Based on factors influencing patient participation identified through a systematic literature review and clinical experience, a structured questionnaire was developed (see Supplementary File 1 for details).

#### General information questionnaire

The General Information Questionnaire was developed by the research team to collect data on participants’ demographic and background characteristics, including gender, age, educational level, self-perceived knowledge of one’s diagnosed condition, self-rated health status, marital status, living arrangement, monthly per capita household income, perceived financial burden of medical expenses, satisfaction with communication with healthcare providers, initiative in communicating with other patients about illness, work experience in the healthcare sector, employment status, religious affiliation, and place of residence. Example item: “How would you rate your overall health status?”

#### Patient Participation scale (PPS)

The Patient Participation Scale (PPS), developed by Song and Kim in 2023, was designed to assess the degree of patient participation in healthcare [31]. It comprises 21 items across four dimensions: *sharing of information and knowledge*, *partaking in the decision-making process*, *performing proactive self-management activities*, and *establishing mutual trust relationship*. Each item is rated on a 5-point Likert scale, yielding a total score ranging from 21 to 105, with higher scores indicating greater patient participation. Example item: “I tell the healthcare provider about my current condition and symptoms in detail.” The scale was translated into Chinese by the research team using a culturally adapted version of Brislin’s translation model [32], and its structure was consistent with the original version. The Chinese version demonstrated good reliability and validity [33], with a Cronbach’s α of 0.929 in the current study.

#### Patient Participation Competence Scale (PPCS)

The Patient Participation Competence Scale (PPCS) was adapted by Chinese scholar Xiang, based on the Patient Participation Competence Scale by Giesler and Weis [34], to assess patients’ competence in participating in healthcare. It consists of 8 items rated on a 5-point Likert scale. Total scores range from 8 to 40, with higher scores reflecting greater participation competence. Example item: “I can proactively express my needs and expectations to the doctor.” The Chinese version of the scale demonstrated good reliability and validity. In this study, the Cronbach’s α was 0.918.

#### Patient Participation Attitude Scale (PPAS)

The Patient Participation Attitude Scale (PPAS) was adapted by Chinese scholar Shen [35], based on the questionnaire developed by Arnetz et al. [36], to assess patients’ attitudes toward participation in healthcare. It consists of 6 items rated on a 5-point Likert scale. Total scores range from 6 to 30, with higher scores indicating more positive attitudes toward patient participation. Example item: “It is important for patients to be involved in decisions about their own treatment and care.” The Chinese version of the scale demonstrated good reliability and validity. In this study, the Cronbach’s α was 0.859.

#### Facilitation of Patient Involvement Scale (FPI)

The Facilitation of Patient Involvement Scale (FPI) was developed by Martin et al. [37] and translated into Chinese by Huang [38] to assess patients’ perceived involvement facilitated by healthcare professionals. It consists of 9 items within a single dimension, rated on a 6-point Likert scale. Total scores range from 6 to 54, with higher scores indicating a greater perceived degree of facilitation for patient participation. Example item: “My doctor provides me with all the information I need to make appropriate decisions.” The Chinese version of the scale demonstrated good reliability and validity. In this study, the Cronbach’s α was 0.894.

#### Family Adaption Partnership Growth Affection and Resolve Index (APGAR)

The Family Adaption Partnership Growth Affection and Resolve Index(APGAR) was developed by Smilkstein [39] and translated into Chinese by Lv et al. [40] to assess individuals’ perceptions of family support. The scale measures five dimensions—adaptability, partnership, growth, affection, and resolve—through five items. Each item is scored on a 3-point scale (0–2), yielding a total score ranging from 0 to 10. Higher scores indicate greater perceived family support and satisfaction with family functioning. Example item: “When I encounter difficulties, I can get satisfactory help from my family.” The Chinese version of the scale demonstrated good reliability and validity. In this study, the Cronbach’s α was 0.852.

#### Mac Arthur Scale of Subjective Social Status (SSS)

The Mac Arthur Scale of Subjective Social Status (SSS) was developed by Goodman et al. [41] and translated into Chinese by Chen et al. [42]. It is currently the most widely used instruments for assessing subjective perceptions of social standing. The scale uses a self-anchoring ladder diagram with ten rungs, each representing a different level of the social hierarchy. Participants are asked to place themselves on the rung that best reflects their perceived social status within society. Example item: “Please mark the step that best represents your social position.” In the Chinese version, the ladder is converted into a numerical scoring system ranging from 1 (lowest social status) to 10 (highest). Higher scores indicate a higher level of perceived subjective social status. In this study, the Cronbach’s α was 0.827.

### Data collection

The patient screening and data collection procedures were carried out by a team of three researchers who had received standardized training, ensuring the rigor and consistency of the process. Prior to data collection, the study objectives and questionnaire content were explained to participants using standardized language to ensure full understanding. Participants were informed that they could withdraw from the study at any point without consequence. All questionnaires were collected on-site and carefully reviewed. Those with incomplete, inconsistent, or repetitive responses were excluded after rigorous evaluation by the research team.

### Statistical analysis

Data were double-entered and cross-validated using EpiData software to ensure accuracy. Statistical analyses were conducted using SPSS 26.0 and Stata 17.0, with the significance level set at *p* < 0.05. Categorical variables were summarized as frequencies and percentages, while continuous variables were presented as means ± standard deviations. Independent-sample *t*-tests and one-way analysis of variance (ANOVA) were used to examine the effects of demographic variables on patient participation. Pearson correlation analysis was used to assess the relationships between patient participation and participation competence, participation attitude, facilitation of patient involvement, family APGAR scores, and subjective socioeconomic status. Hierarchical linear regression was performed to identify the determinants of patient participation. Shapley value decomposition based on regression analysis was used to quantify the relative contributions of each factor at different ecological levels.

## Results

A total of 573 patients were initially recruited for the study. Of these, 32 declined participation, 14 withdrew during questionnaire completion for personal reasons, and 7 were excluded due to duplicate or incomplete responses. Therefore, 520 valid questionnaires were included in the final analysis.

### Characteristics of the study participants

The study included 520 participants aged 23 to 93 years, with a mean age of 56.31 years (SD = 14.95). Additional demographic characteristics are detailed in Table 1.

**Table 1:** Demographic characteristics of study participants and patient participation across micro-, meso-, and macro-level categorical variables (*n* = 520)

Variables	*n* (%)	Patient participation score (Mean ± SD)	*t*-/*F*-value	*p*-value
Microsystem				
Gender			1.434^a^	0.152
Male	239 (45.96)	77.39 ± 12.56		
Female	281 (54.04)	78.97 ± 12.47		
Age (years)			7.165^a^	< 0.001^*^
< 60	309 (59.42)	81.35 ± 11.42		
≥ 60	211 (40.58)	73.70 ± 12.70		
Educational level			63.542^b^	< 0.001^*^
Primary school or below	97 (18.65)	69.69 ± 10.64		
Junior high school	151 (29.04)	73.02 ± 11.70		
Senior high school or technical secondary school	113 (21.73)	81.83 ± 10.69		
Junior college, bachelor’s degree or above	159 (30.58)	85.88 ± 9.81		
Self-perceived knowledge of one’s diagnosed condition			32.455^b^	< 0.001^*^
Completely unaware	21 (4.04)	69.38 ± 11.53		
Slightly aware	234 (45.00)	73.71 ± 11.96		
Moderately aware	246 (47.31)	82.54 ± 11.25		
Highly aware	19 (3.65)	88.32 ± 9.10		
Self-rated health status			17.748^b^	< 0.001^*^
Excellent	55 (10.58)	82.73 ± 10.81		
Good	254 (48.85)	80.91 ± 11.50		
Fair	139 (26.73)	76.47 ± 12.26		
Poor	53 (10.19)	69.72 ± 12.48		
Very poor	19 (3.65)	66.42 ± 12.67		
Mesosystem				
Marital status			0.930^a^	0.353
Single (including never married, divorced, or widowed)	57 (10.96)	79.70 ± 11.31		
Married or cohabiting	463 (89.04)	78.07 ± 12.66		
Living arrangement			0.346^a^	0.729
Living alone	33 (6.35)	77.52 ± 14.72		
Not living alone	487 (93.65)	78.30 ± 12.37		
Monthly per capita household income (RMB)			22.052^b^	< 0.001^*^
<2000	88 (16.92)	72.49 ± 12.75		
2000 ~ 4999	255 (49.04)	76.63 ± 12.37		
5000 ~ 7999	127 (24.42)	82.04 ± 10.74		
≥ 8000	50 (9.62)	87.00 ± 9.62		
Perceived financial burden of medical expenses			25.396^b^	< 0.001^*^
No burden	67 (12.88)	87.63 ± 9.44		
Slight burden	294 (56.54)	78.74 ± 11.51		
Moderate burden	117 (22.50)	74.68 ± 12.90		
Severe burden	42 (8.08)	69.79 ± 12.93		
Satisfaction with communication with healthcare providers			6.595^a^	< 0.001^*^
Satisfied	398 (76.54)	80.17 ± 11.95		
Not satisfied	122 (23.46)	71.96 ± 12.33		
Initiative in communicating with other patients about illness			3.066^a^	0.002^*^
Yes	394 (75.77)	79.19 (12.41)		
No	126 (24.23)	75.29 (12.45)		
Work experience in the healthcare sector			4.557^a^	< 0.001^*^
Yes	246 (47.31)	80.84 ± 11.81		
No	274 (52.69)	75.92 ± 12.70		
Employment status			5.467^b^	0.004^*^
Unemployed	170 (32.69)	75.71 ± 12.07		
Employed	216 (41.54)	79.79 ± 12.27		
Retired	134 (25.77)	78.98 ± 13.07		
Macrosystem				
Religious affiliation			0.995^a^	0.320
Yes	28 (5.38)	80.54 ± 11.08		
No	492 (94.62)	78.12 ± 12.59		
Place of residence			8.764^a^	< 0.001^*^
Rural	169 (32.50)	71.77 ± 12.44		
Urban or township	351 (67.50)	81.36 ± 11.32		

Abbreviations: ^a^ *t*-test.; ^b^ *F*-test.; ^*^ *p* < 0.05

### Description of patient participation

The total patient participation score was 78.25 ± 12.52, with a mean item score of 3.56 ± 0.57. Among the four dimensions, the highest mean item score was observed in *establishing mutual trust relationships* (3.91 ± 0.76), followed by *partaking in the decision-making process* (3.86 ± 0.80), *sharing of information and knowledge* (3.81 ± 0.71), and the lowest in *performing proactive self-management activities* (3.48 ± 0.75). At the item level, the highest-scoring item was “I tell the healthcare providers (HCPs) about my current condition and symptoms in detail,” whereas the lowest was “I monitor whether the HCP identifies the patient before performing examination, medication, or tests.” Detailed results of dimension-level and item-level scores are presented in Tables 2 and 3.

**Table 2:** Patient participation scores by dimension (*n* = 520)

Dimension	Number of items	Maximum score	Total score (Mean ± SD)	Mean item score (Mean ± SD)
Total score	21	105	78.25 ± 12.52	3.56 ± 0.57
Establishing mutual trust relationships	4	20	15.64 ± 3.06	3.91 ± 0.76
Partaking in the decision-making process	2	10	7.72 ± 1.60	3.86 ± 0.80
Sharing of information and knowledge	8	40	30.50 ± 5.69	3.81 ± 0.71
Performing proactive self-management activities	7	35	24.38 ± 5.23	3.48 ± 0.75

**Table 3:** Patient participation scores by item (*n* = 520)

Ranking	Item	Dimension	Item score (Mean ± SD
Top 5 items	1 I tell the HCP about my current condition and symptoms in detail.	Sharing of information and knowledge	4.04 ± 0.90
	3 I inform the HCP of specific information to refer to for my treatment.	Sharing of information and knowledge	3.98 ± 0.89
	20 I think the HCP respects me.	Establishing mutual trust relationships	3.95 ± 0.86
	19 I believe that my HCP is well aware of my condition and treatment progress.	Establishing mutual trust relationships	3.93 ± 0.85
	9 I decide on the treatment method recommended by the HCP after referring to my current condition and the opinions of my family or acquaintances.	Partaking in the decision-making process	3.93 ± 0.84
Bottom 5 items	13 I check if my treatment proceeds according to the guided schedule.	Performing proactive self-management activities	3.52 ± 0.95
	15 I comply with the fall prevention activities given by the hospital.	Performing proactive self-management activities	3.45 ± 0.96
	14 I comply with the infection prevention activities, such as hand washing (hand hygiene).	Performing proactive self-management activities	3.39 ± 0.97
	17 I monitor whether the HCP washes their hands (hand hygiene) before performing any tests, medications, or treatments.	Performing proactive self-management activities	3.27 ± 1.03
	16 I monitor whether the HCP identifies the patient before performing examination, medication, or tests.	Performing proactive self-management activities	3.25 ± 0.99

### Univariate analysis of patient participation

Differences in patient participation across categorical variables at the micro-, meso-, and macro-system levels were analyzed using independent samples *t*-tests and one-way ANOVA. Significant differences were observed in the microsystem variables including age, educational level, self-perceived knowledge of one’s diagnosed condition, and self-rated health status (*p* < 0.05). In the mesosystem, monthly per capita household income, perceived financial burden of medical expenses, satisfaction with communication with healthcare providers, initiative in communicating with other patients about illness, work experience in the healthcare sector, and employment status were also statistically significant (*p* < 0.05). At the macrosystem, place of residence was significantly associated with patient participation (*p* < 0.05), as detailed in Table 1.

### Correlation between participation competence, participation attitude, facilitation of patient participation, family support, subjective socioeconomic status, and patient participation

Pearson correlation analysis revealed that participation competence, participation attitude, facilitation of patient participation, family support, and subjective socioeconomic status were all positively associated with patient participation (*p* < 0.01). The correlation coefficients ranged from 0.344 to 0.550, indicating moderate positive relationships (Table 4).

**Table 4:** Correlation between patient participation and micro-, meso-, and macro-level continuous variables (*n* = 520)

Variables	Total score (Mean ± SD)	Correlation with patient participation	
		*r*	*p*-value
Microsystem			
Participation competence	27.62 ± 6.30	0.550	< 0.001
Participation attitude	23.50 ± 4.48	0.500	< 0.001
Mesosystem			
Facilitation of patient participation	40.74 ± 6.50	0.422	< 0.001
Family support	2.76 ± 2.34	0.377	< 0.001
Macrosystem			
Subjective socioeconomic status	9.89 ± 3.40	0.344	< 0.001

### Hierarchical regression analysis of patient participation

A hierarchical linear regression analysis was conducted with patient participation score as the dependent variable. Independent variables that showed statistical significance (*p* < 0.05) in both univariate and correlation analyses were included. Variables from the microsystem were entered into Model 1. Mesosystem variables were added in Model 2, followed by macrosystem variables in Model 3. Multicollinearity diagnostics indicated no multicollinearity among the independent variables, with Variance Inflation Factor (VIF) values ranging from 1.028 to 1.869.

In Model 1, microsystem variables including age, educational level, self-perceived knowledge of one’s diagnosed condition, self-rated health status, participation competence, and participation attitude were significantly associated with patient participation (*p* < 0.05). In Model 2, after controlling for microsystem variables, mesosystem factors including perceived financial burden of medical expenses, satisfaction with communication with healthcare providers, facilitation of patient participation, and family support were also significantly associated with patient participation (*p* < 0.05). In Model 3, after controlling for both microsystem and mesosystem variables, macrosystem factors including place of residence and subjective socioeconomic status remained significantly associated with patient participation (*p* < 0.05). Further details are available in Table 5.

### Shapley value decomposition of factors influencing patient participation

Shapley value decomposition, originally derived from Shorrocks’ natural decomposition framework and cooperative game theory, allows for the decomposition of additive explanatory power in regression models to quantify the relative importance of each predictor [43]. In this study, the method was applied to further explore the contributions of variables from different system levels (micro, meso, and macro) to patient participation, based on the final hierarchical regression model.

**Fig. 2:**
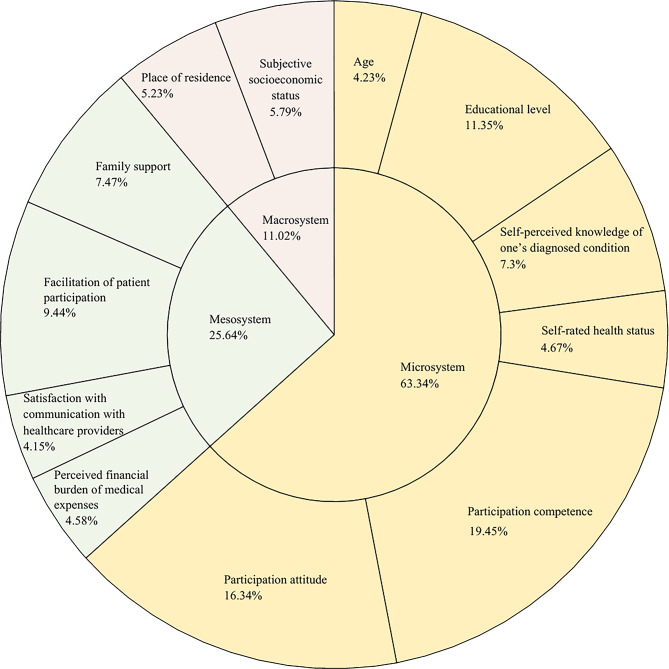
Shapley value decomposition of factors influencing patient participation

Shapley value decomposition was conducted using Stata 17.0. The results revealed that, within the framework of the Social Ecological Model, the microsystem contributed the most to explaining patient participation (63.34%), followed by the mesosystem (25.64%) and macrosystem (11.02%). Among all predictors, participation competence had the highest explanatory contribution (19.45%), while satisfaction with communication with healthcare providers had the lowest (4.15%). Further details are presented in Fig. 2.

## Discussion

**Table 5:** Hierarchical regression analysis of patient participation

Variables	Model 1					Model 2					Model 3				
	B(95%CI)	SE	β	*t*	*P*	B(95%CI)	SE	β	*t*	*P*	B(95%CI)	SE	β	*t*	*P*
Age	−3.854 (−5.405,−2.304)	0.789	−0.151	−4.883	< 0.001	−2.396 (−3.874,−0.918)	0.752	−0.094	−3.184	0.002	−2.323 (−3.785,−0.860)	0.744	−0.091	−3.120	0.002
Educational level	2.235 (1.458, 3.013)	0.396	0.197	5.647	< 0.001	1.527 (0.736, 2.317)	0.402	0.135	3.793	< 0.001	1.334 (0.522, 2.146)	0.413	0.118	3.229	0.001
Self-perceived knowledge of one’s diagnosed condition	2.981 (1.727, 4.236)	0.638	0.151	4.669	< 0.001	2.551 (1.381, 3.721)	0.596	0.130	4.284	< 0.001	2.375 (1.214, 3.536)	0.591	0.121	4.018	< 0.001
Self-rated health status	−0.984 (−1.829,−0.138)	0.430	−0.074	−2.286	0.023	−0.877 (−1.680,−0.074)	0.409	−0.066	−2.146	0.032	−0.922 (−1.716,−0.128)	0.404	−0.069	−2.281	0.023
Participation competence	0.661 (0.536, 0.786)	0.063	0.332	10.418	< 0.001	0.581 (0.463, 0.699)	0.060	0.292	9.689	< 0.001	0.537 (0.417, 0.656)	0.061	0.270	8.829	< 0.001
Participation attitude	0.857 (0.687, 1.027)	0.086	0.307	9.916	< 0.001	0.708 (0.546, 0.869)	0.082	0.253	8.598	< 0.001	0.671 (0.510, 0.833)	0.082	0.240	8.191	< 0.001
Monthly per capita household income	—	—	—	—	—	−0.223 (−1.258, 0.811)	0.527	−0.015	−0.424	0.672	−0.313 (−1.337, 0.711)	0.521	−0.021	−0.600	0.549
Perceived financial burden of medical expenses	—	—	—	—	—	−1.259 (−2.333,−0.184)	0.547	−0.079	−2.301	0.022	−1.171 (−2.235,−0.107)	0.542	−0.073	−2.162	0.031
Satisfaction with communication with healthcare providers	—	—	—	—	—	−3.001 (−4.653,−1.349)	0.841	−0.102	−3.570	< 0.001	−2.907 (−4.541,−1.273)	0.832	−0.098	−3.495	0.001
Initiative in communicating with other patients about illness	—	—	—	—	—	0.313 (−1.291, 1.916)	0.816	0.011	0.383	0.702	0.331 (−1.255, 1.916)	0.807	0.011	0.410	0.682
Employment status	—	—	—	—	—	0.663 (−0.217, 1.544)	0.448	0.040	1.480	0.140	0.599 (−0.272, 1.470)	0.444	0.036	1.351	0.177
Work experience in the healthcare sector	—	—	—	—	—	−0.399 (−1.790, 0.992)	0.708	−0.016	−0.563	0.574	−0.327 (−1.711, 1.057)	0.704	−0.013	−0.465	0.642
Facilitation of patient participation	—	—	—	—	—	0.338 (0.225, 0.452)	0.058	0.176	5.868	< 0.001	0.297 (0.182, 0.411)	0.058	0.154	5.085	< 0.001
Family support	—	—	—	—	—	0.650 (0.340, 0.959)	0.158	0.121	4.124	< 0.001	0.652 (0.346, 0.958)	0.156	0.122	4.186	< 0.001
Place of residence	—	—	—	—	—	—	—	—	—	—	1.878 (0.282, 3.474)	0.812	0.070	2.312	0.021
Subjective socioeconomic status	—	—	—	—	—	—	—	—	—	—	0.319 (0.109, 0.530)	0.107	0.087	2.976	0.003
R^2^	0.567					0.634					0.643				
∆R^2^	0.567					0.067					0.010				
F	111.819^**^					62.424^**^					56.712^**^				

Abbreviations: ^*^P < 0.05; ^**^P < 0.001

This cross-sectional survey assessed patient participation in healthcare in North China. Based on Social Ecological Model, the study systematically explored factors influencing patient participation and revealed how factors from different system levels affect it. Overall, the influence of these systems on patient participation ranked in descending order as microsystem, mesosystem, and macrosystem. Regarding specific variables, their relative contributions from highest to lowest were: participation competence, participation attitude, educational level, facilitation of patient participation, family support, self-perceived knowledge of one’s diagnosed condition, subjective socioeconomic status, place of residence, self-rated health status, perceived financial burden of medical expenses, age, and satisfaction with communication with healthcare providers.

Moreover, the convenience sampling strategy used in this study was closely tied to the data collection process conducted within tertiary hospitals, where patients with higher health literacy or more active engagement were more likely to participate. This may have introduced selection bias, leading to an underrepresentation of individuals with lower participation willingness or limited access to tertiary care. Therefore, both the sampling approach and data collection context may have influenced the observed level of patient participation and its generalizability.

### Analysis of the current status of patient participation

The mean total score of patient participation in healthcare was 78.25 ± 12.52, with a mean item score of 3.56 ± 0.57, indicating a moderate level of participation compared to the scale’s median of 3. Among the four dimensions, *establishing mutual trust relationships* scored highest. In contrast, *performing proactive self-management activities* scored lowest. At the item level, the highest scores were for “I tell the HCP about my current condition and symptoms in detail” and “I inform the HCP of specific information to refer to for my treatment”. The lowest were “I monitor whether the HCP identifies the patient.” and “I comply with infection prevention activities.”. Previous studies similarly found that patients are more willing to ask about conditions and treatment but less likely to question hygiene practices, possibly to avoid confrontation [44, 45]. These findings indicate that participation remains focused on basic information sharing, with inadequate involvement in safety and self-management. Therefore, healthcare institutions should strengthen patient education regarding patients’ rights and responsibilities to enhance their awareness of their roles in the healthcare process, especially in areas related to treatment safety and self-management. For example, implementing regular patient safety education, publicizing safety monitoring procedures, and fostering a supportive communication environment may facilitate more active patient participation.

### Analysis of factors influencing patient participation

#### Microsystem factors

Microsystem factors contributed the most to patient participation, accounting for 63.34% of the explained variance. Among the six microsystem-level variables, the contributions to patient participation ranked as follows: participation competence (19.45%), participation attitude (16.34%), educational level (11.35%), self-perceived knowledge of one’s diagnosed condition (7.3%), self-rated health status (4.67%), and age (4.23%).

Our study revealed that among the various factors influencing patient participation, participation competence and participation attitude contributed the most. A qualitative meta-summary also identified insufficient competence and low self-efficacy as major barriers [46]. According to the Knowledge-Attitude-Practice (KAP) theory, behavior change progresses from knowledge to belief, and ultimately to action [47]. In this study, participation competence refers to patients’ abilities to obtain medical information, communicate with providers, seek support, and make care decisions. Participation attitude reflects patients’ perceived value and their willingness to engage. These two factors are interrelated and mutually reinforcing. Patients with competence but low motivation may avoid participation, while those with a positive attitude but inadequate skills may struggle to engage effectively. Therefore, intervention strategies should adopt a “dual-engine” approach, enhancing both competence and attitude. Patient education and skill training can strengthen competence, while improved communication and a supportive environment can boost motivation and self-efficacy. Future research should explore effective interventions for both dimensions, with attention to differences between outpatient and inpatient populations, to guide more targeted and personalized participation strategies.

The study indicated that patients with higher educational levels tend to demonstrate greater participation in healthcare, which aligns with previous findings [48, 49]. This may be because more educated patients typically have stronger learning and information-processing abilities, allowing them to better understand medical information, diagnostic results, and treatment plans. They are also more skilled at using diverse sources—such as the internet and medical literature—to acquire disease-related knowledge, which enhances their confidence in facing complex decisions and encourages active communication with healthcare providers [50]. Additionally, highly educated patients often exhibit stronger critical thinking and are more inclined to evaluate treatment options before accepting medical advice [21], leading to deeper discussions with physicians. In contrast, patients with lower education levels may have limited access to and comprehension of medical information, making them more likely to passively accept medical decisions and show lower participation. Therefore, healthcare professionals should provide tailored support and guidance to patients with less education to help them better understand medical information and enhance their participation in care.

This study found a positive association between patients’ understanding of their diagnosed conditions and their level of participation in healthcare. Prior research has similarly identified insufficient disease-related knowledge as a major barrier to patient engagement [49, 51, 52]. For example, patients with a better understanding of atrial fibrillation were more likely to take an active role in treatment decisions regarding catheter ablation [53]. When patients have a clear understanding of their illness, they are better equipped to reduce information asymmetry with providers, comprehend treatment plans, recognize critical symptoms, and engage in both decision-making and self-management. In contrast, limited disease understanding can hinder patients’ ability to follow medical information and may lead them to perceive themselves as unqualified to participate [46]. Although abundant medical content is available online, patients often struggle to distinguish credible information from misinformation. Moreover, most resources remain overly simplistic and fail to meet patients’ needs for in-depth knowledge [54]. Therefore, healthcare providers should deliver personalized education tailored to patients’ conditions.

This study found that patients with better self-rated health status demonstrated higher levels of participation in healthcare. Consistent with this, Bosio et al. [48] reported that individuals with fewer than three comorbidities were more actively involved in care compared to those with more chronic conditions. Poor self-perceived health is often accompanied by negative emotions such as anxiety and depression, which may reduce patients’ motivation to seek medical information and participate in decision-making. Additionally, the complexity of managing multiple illnesses and the ongoing treatment burden can lead to feelings of helplessness and diminished confidence in self-management. Physical limitations and fatigue may further hinder their ability to communicate effectively and engage in care, making them more reliant on healthcare providers. In contrast, patients who perceive themselves as healthy tend to have better physical functioning and a more positive mental outlook, enabling more active participation in treatment decisions and health management. They are generally more confident, have higher expectations for care outcomes, and are more willing to communicate with physicians and express preferences. These findings highlight the need for targeted support for patients with poor self-rated health—particularly in addressing emotional well-being and cognitive burden. Interventions such as psychological counseling and emotional support may help restore their confidence in managing their health and progressively enhance their level of participation.

This study found that patients under 60 years of age exhibited higher levels of participation in healthcare. This finding is consistent with previous research showing that younger patients are more involved in shared decision-making [49, 55]. Several factors may explain this trend. Younger individuals often juggle multiple roles—such as caregiving for children or elderly family members and fulfilling work responsibilities—which may lead to higher expectations for treatment outcomes and stronger motivation to engage in care. Additionally, they are generally more proficient in accessing, understanding, and utilizing health-related information via the Internet and other sources. These capabilities enhance their disease understanding and self-management. In contrast, older adults are more likely to experience physiological and cognitive decline, which may hinder their ability to comprehend medical information and reduce expectations for treatment outcomes. As a result, their participation tends to be lower. Moreover, age-related biases and marginalization in clinical settings may deprive them of opportunities to express preferences or result in passive acceptance of decisions made by healthcare professionals or family members [56]. Therefore, healthcare providers should pay greater attention to the needs of older patients by offering decision-making support based on their cognitive and communication characteristics. Actively fostering an inclusive and age-friendly clinical environment is also essential to promote equitable participation. Additionally, potential selection and measurement biases related to self-reported data are discussed in the limitations section.

#### Mesosystem factors

The results showed that mesosystem factors explained 25.64% of the variance in patient participation, representing a moderate contribution. Among the four mesosystem-level variables, their contributions to participation, in descending order, were: facilitation of patient participation (9.44%), family support (7.47%), perceived financial burden of medical expenses (4.58%), and satisfaction with communication with healthcare providers (4.15%).

The findings of this study indicate that patients who perceive stronger facilitation from healthcare professionals exhibit higher levels of participation. Previous research supports this, showing that physicians’ encouraging communication promotes patient involvement [49], and that perceived support is significantly associated with greater engagement in treatment decisions [57]. In the context of traditional Chinese culture, patients and their families often place strong trust in the authority of healthcare professionals, who serve not only as primary sources of medical information but also as key providers of emotional support [58, 59]. Therefore, clinical practice should prioritize encouraging patient participation and delivering condition-specific, individualized information—rather than relying on generalized advice [60]. Despite widespread recognition of its importance, promoting patient participation remains challenging. Barriers such as time constraints, limited staffing, and low confidence or self-efficacy among healthcare providers can hinder its implementation [11, 49, 54]. To address these challenges, healthcare systems should offer structured training and practical tools—such as shared decision-making aids—to support more personalized and collaborative care delivery [61, 62]. Additionally, hospital administrators must ensure institutional support by optimizing workloads and enabling sufficient time for meaningful patient-provider communication.

This study found that higher levels of family support were significantly associated with greater patient participation in healthcare. Previous research has similarly identified family support as a critical facilitator of patient engagement [46, 49]. Family members contribute across multiple domains. First, they provide informational support by helping patients clarify medical histories, record key information, and better understand diagnostic recommendations and treatment instructions—particularly important for older adults. Second, emotional support plays a vital role. Companionship and reassurance from family can stabilize patients’ emotional state, foster a positive cognitive outlook, alleviate psychological distress, and boost confidence in managing illness, thereby promoting more active engagement [63]. Third, financial support from family is often essential for ensuring continuity of treatment and meaningful participation in decision-making. Therefore, healthcare professionals should actively involve and guide family members in the caregiving process, emphasizing their roles in information exchange, emotional reassurance, and financial assistance.

This study found that patients experiencing a lower financial burden from medical expenses exhibited higher levels of participation in healthcare. Previous research has shown that individuals covered by rural medical insurance tend to be less engaged than those with urban insurance, possibly due to lower reimbursement rates and greater out-of-pocket costs for rural residents [64]. The degree of financial burden can significantly influence patients’ autonomy in making healthcare decisions. Those under less economic pressure often have more flexibility in choosing providers, medications, and treatment plans. They are also more likely to seek information proactively, compare alternatives, and consider advanced therapeutic options that align with their personal needs and clinical circumstances. In contrast, patients facing high medical costs may be restricted in their treatment choices and rely passively on physicians’ decisions. This can lead to disengagement or even negative attitudes toward participation [65]. Therefore, healthcare professionals should consider patients’ financial situations during clinical encounters and assist them in accessing social support mechanisms, such as insurance programs, financial aid, and philanthropic healthcare initiatives. Reducing patients’ financial burden may enhance their participation.

This study found that patients who were satisfied with communication with healthcare providers demonstrated higher levels of participation in healthcare. Healthcare professionals are the primary source of disease-related information, and effective communication is a key facilitator of patient engagement [66]. However, studies have shown that physicians often overestimate their communication skills. For instance, one study reported that while 75% of orthopedic surgeons believed their communication was satisfactory, only 21% of patients agreed. Most patients expressed a desire for more in-depth and empathetic interactions with their doctors [67]. To improve patient participation, healthcare professionals must develop not only clinical competencies but also communication skills. Critical information should be conveyed in ways that are clear and accessible to patients. Actively listening to patients’ concerns, incorporating their feedback, and respecting their preferences are essential for building strong therapeutic relationships. Furthermore, sufficient provider–patient communication time has been identified as a prerequisite for effective patient participation [11]. Yet, current heavy clinical workloads often limit the time available for such communication [52]. Therefore, hospital administrators should optimize staffing and scheduling to ensure adequate time for meaningful interactions. In parallel, targeted training—such as the use of structured communication strategies like the “teach-back” method [68]—can help systematically enhance providers’ communication skills and further promote patient participation.

#### Macrosystem factors

The results showed that macrosystem factors had the least explanatory power for patient participation, accounting for 11.02% of the total explained variance. Among the two macrosystem-level variables, subjective socioeconomic status contributed slightly more (5.79%) than place of residence (5.23%).

The results of this study indicate that patients with higher subjective socioeconomic status (SES) tend to exhibit greater participation in healthcare. Previous research has shown that SES influences health-related attitudes and behaviors [69]. Individuals from socially disadvantaged groups are generally less likely to participate in healthcare [70], whereas those with higher incomes tend to be more actively engaged [53]. This association may be explained by several factors. First, patients with higher subjective SES often possess stronger information-processing abilities, enabling them to better comprehend medical information, assess treatment options, and communicate effectively with healthcare providers [71, 72]. Second, they tend to display greater confidence and assertiveness in medical settings, making it easier to express preferences and advocate for themselves. In contrast, individuals with lower SES may believe they are “unqualified” or “incapable” of participating in their own care [73]. Therefore, efforts to promote patient participation must include targeted support for socioeconomically disadvantaged groups. Stratified interventions—such as improving access to communication channels and healthcare resources—are essential for fostering more equitable participation.

This study found that patients residing in rural areas exhibited significantly lower levels of participation in healthcare compared to those in urban or suburban regions. Two primary factors may account for this disparity. First, rural residents often have limited access to reliable medical information and frequently rely on grassroots providers or informal sources such as family members. These channels tend to be delayed and incomplete, hindering patients’ understanding of their condition and limiting proactive engagement. Second, the uneven distribution of healthcare resources between urban and rural areas exacerbates the issue. Rural communities often face shortages of medical institutions and qualified professionals, which restricts access to consistent, high-quality care and reduces opportunities for active participation. To address this imbalance, it is essential to strengthen primary healthcare infrastructure, extend the reach of health education in rural communities, and expand diversified and accessible channels for health information dissemination. These measures can help improve rural patients’ understanding of medical issues and enhance their ability to participate meaningfully in healthcare.

Despite its valuable findings, this study has several limitations that should be addressed in future research. First, it employed a purely quantitative approach without integrating qualitative methods, such as in-depth interviews, to explore the underlying mechanisms of patient participation. This limits the understanding of patients’ subjective experiences and behavioral motivations. Future studies should consider a mixed-methods design to enrich both analytical depth and contextual interpretation, providing more robust theoretical insights and practical guidance for intervention development. Second, potential selection and measurement biases should be acknowledged. Because data were collected through self-reported questionnaires administered in tertiary hospitals, individuals with lower health literacy, limited cognitive ability, or reduced willingness to engage may have been underrepresented. These groups might experience distinct participation barriers that were not fully captured in this study. Furthermore, self-assessment measures are inherently subject to response bias, as participants may overestimate or underestimate their engagement due to social desirability or misinterpretation of items. Future research should therefore employ more diverse sampling strategies and combine self-report data with observational or qualitative methods to enhance the validity and inclusiveness of findings. Third, the measurement tools used lacked refined instruments for assessing macro-level influences such as cultural background and policy context. This may have restricted the evaluation of macrosystemic factors, reducing the overall explanatory power and comprehensiveness of the findings. Fourth, due to its cross-sectional design, the study cannot establish causal relationships between influencing factors and patient participation. Longitudinal or experimental studies are recommended to better examine causal pathways and dynamic changes over time. Finally, several demographic variables (e.g., marital status, living arrangement, and religious affiliation) showed limited variability, as most participants were married, living with family, and reported no religious affiliation. This restricted variability may have reduced the explanatory power of these factors in the statistical analyses.

## Conclusions

This study assessed the level of patient participation in healthcare and identified its determinants based on the Social Ecological Model. Patient participation was found to be moderate, with the microsystem exerting the greatest influence, followed by the mesosystem and macrosystem. Participation competence and attitude were the most influential factors, while educational level, facilitation by healthcare providers, family support, health knowledge, and socioeconomic status also contributed. These findings provide an empirical basis for developing targeted, multilevel strategies to enhance patient engagement. Clinical interventions should focus on vulnerable populations—such as those with lower education, rural residence, poorer health, higher financial burden, and older age—by strengthening participation competence, fostering positive attitudes, and improving disease understanding. Family involvement remains vital in offering emotional and informational support, and healthcare institutions should promote effective provider–patient communication and equitable resource allocation. Integrating the concept of patient participation into medical and nursing education may further reinforce shared decision-making and patient-centered care. Collectively, these actions can advance a more equitable and participatory healthcare system aligned with the goals of people-centered reform and health equity.

## Supplementary Information

Below is the link to the electronic supplementary material.

Supplementary Material 1

## Data Availability

Data are available upon justified request to the correspondence author.
