# Supplementary material for: Patient participation and its determinants based on the Social Ecological Model: a cross-sectional study in North China
Source: BMC Health Serv Res. 2025 Nov 21;25:1541. doi: 10.1186/s12913-025-13795-2 (PMC12659090; doi:10.1186/s12913-025-13795-2)
Supplement: Supplementary file 1 — Supplementary Material 1 [file 12913_2025_13795_MOESM1_ESM.docx]

**Questionnaire on the Current Situation and Influencing Factors of Patient Participation**

Dear patient,

Greetings! We sincerely appreciate you taking the time out of your busy schedule to participate in our survey. Patient participation in healthcare can enhance self-management abilities and improve health outcomes. This questionnaire aims to assess the level of your participation in healthcare during medical visits. Your responses will be anonymous, and the results will be used solely for clinical scientific research. All personal information will remain strictly confidential, so please feel assured when completing the questionnaire. Thank you once again for your support and cooperation. We wish you a speedy recovery!

**General Information Questionnaire**

1. Your gender is:
2. Male B. Female
3. Your age is:
4. ＜60 years B. ≥60 years
5. Do you have a religious affiliation?
6. Yes B. No
7. Your educational level:
8. Primary school or below B. Junior high school C. Senior high school or technical secondary school D. Junior college, bachelor’s degree or above
9. Your marital status:

A. Single (including never married, divorced, or widowed) B. Married or cohabiting

1. Your place of residence:
2. Rural B. Urban or township
3. Your living arrangement:
4. Living alone B. Not living alone
5. Your employment status:

A. Unemployed B. Employed C. Retired

1. Do you have work experience in the healthcare sector?

A. Yes B. No

1. Your monthly per capita household income:
2. ＜2000 yuan B. 2000~4999 yuan C. 5000~7999 yuan D. ＞8000 yuan
3. Your perceived financial burden of medical expenses:
4. No burden B. Slight burden C. Moderate burden D. Severe burden
5. Your self-perceived knowledge of one’s diagnosed condition:
6. Completely unaware B. Slightly aware C. Moderately aware D. Highly aware
7. Are you satisfied with communication with healthcare providers?
8. Satisfied B. Not satisfied
9. Do you take the initiative in communicating with other patients about illness?

A. Yes B. No

1. Your self-rated health status:
2. Excellent B. Good C. Fair D. Poor E. Very poor

**Patient Participation Scale**

1. I tell the HCP about my current condition and symptoms in detail.
2. Strongly disagree B. Disagree C. Uncertain D. Agree E. Strongly agree
3. I inform the HCP if new symptoms occur or existing symptoms change.
4. Strongly disagree B. Disagree C. Uncertain D. Agree E. Strongly agree
5. I inform the HCP of specific information to refer to for my treatment.
6. Strongly disagree B. Disagree C. Uncertain D. Agree E. Strongly agree
7. I tell the HCP how I am managing my disease.
8. Strongly disagree B. Disagree C. Uncertain D. Agree E. Strongly agree
9. I check with the HCP whether the information and knowledge I have acquired (food, medications, and treatment methods, etc.) are correct.
10. Strongly disagree B. Disagree C. Uncertain D. Agree E. Strongly agree
11. I ask the HCP any questions I may have about the disease or the treatment.
12. Strongly disagree B. Disagree C. Uncertain D. Agree E. Strongly agree
13. I ask for further explanation if I do not understand the HCP’s explanation.
14. Strongly disagree B. Disagree C. Uncertain D. Agree E. Strongly agree
15. I listen carefully to the HCP’s explanation.
16. Strongly disagree B. Disagree C. Uncertain D. Agree E. Strongly agree
17. I decide on the treatment method recommended by the HCP after referring to my current condition and the opinions of my family or acquaintances.
18. Strongly disagree B. Disagree C. Uncertain D. Agree E. Strongly agree
19. I consult with the HCP if I find a more suitable alternative during the treatment process.
20. Strongly disagree B. Disagree C. Uncertain D. Agree E. Strongly agree
21. I check my vital signs (blood pressure, pulse rate, body temperature, and respiration rate) or test results and compare them with previous results.
22. Strongly disagree B. Disagree C. Uncertain D. Agree E. Strongly agree
23. I observe whether new symptoms occur or existing symptoms change.
24. Strongly disagree B. Disagree C. Uncertain D. Agree E. Strongly agree
25. I check if my treatment proceeds according to the guided schedule.
26. Strongly disagree B. Disagree C. Uncertain D. Agree E. Strongly agree
27. I comply with the infection prevention activities, such as hand washing (hand hygiene).
28. Strongly disagree B. Disagree C. Uncertain D. Agree E. Strongly agree
29. I comply with the fall prevention activities given by the hospital.
30. Strongly disagree B. Disagree C. Uncertain D. Agree E. Strongly agree
31. I monitor whether the HCP identifies the patient before performing examination, medication, or tests.
32. Strongly disagree B. Disagree C. Uncertain D. Agree E. Strongly agree
33. I monitor whether the HCP washes their hands (hand hygiene) before performing any tests, medications, or treatments.
34. Strongly disagree B. Disagree C. Uncertain D. Agree E. Strongly agree
35. I trust and follow the expertise and experience of the HCP.
36. Strongly disagree B. Disagree C. Uncertain D. Agree E. Strongly agree
37. I believe that my HCP is well aware of my condition and treatment progress.
38. Strongly disagree B. Disagree C. Uncertain D. Agree E. Strongly agree
39. I think the HCP respects me.
40. Strongly disagree B. Disagree C. Uncertain D. Agree E. Strongly agree
41. I think the HCP listens to me.
42. Strongly disagree B. Disagree C. Uncertain D. Agree E. Strongly agree

**Patient Participation Competence Scale**

1. I can find information about diseases and treatments from books or the Internet.
2. Strongly disagree B. Disagree C. Neutral D. Agree E. Strongly agree
3. I can find some complementary treatment methods (e.g., acupuncture, massage, etc.).
4. Strongly disagree B. Disagree C. Neutral D. Agree E. Strongly agree
5. I can proactively inform the doctor about my medical history, allergies, symptoms, and other relevant information.
6. Strongly disagree B. Disagree C. Neutral D. Agree E. Strongly agree
7. I can proactively express my needs and expectations to the doctor.
8. Strongly disagree B. Disagree C. Neutral D. Agree E. Strongly agree
9. I am capable of discussing examination and treatment plans with the doctor.
10. Strongly disagree B. Disagree C. Neutral D. Agree E. Strongly agree
11. I am capable of choosing between different treatment options (e.g., whether to have surgery, drug therapy, etc.).
12. Strongly disagree B. Disagree C. Neutral D. Agree E. Strongly agree
13. If I am dissatisfied during the treatment process, I will report it to the department head, the hospital administration, or the relevant department.
14. Strongly disagree B. Disagree C. Neutral D. Agree E. Strongly agree
15. In the event of a dispute, I know how to resolve it (whom to report to, knowing the complaint hotline, etc.).
16. Strongly disagree B. Disagree C. Neutral D. Agree E. Strongly agree

**Patient Participation Attitude Scale**

1. It is important for patients to receive clear and understandable information about their treatment.
2. Strongly disagree B. Somewhat disagree C. Uncertain D. Somewhat agree E. Strongly agree
3. It is important for patients to ask questions related to their own condition.
4. Strongly disagree B. Somewhat disagree C. Uncertain D. Somewhat agree E. Strongly agree
5. It is important for patients to express his/her own views.
6. Strongly disagree B. Somewhat disagree C. Uncertain D. Somewhat agree E. Strongly agree
7. It is important for patients to participate in discussions about their own treatment and care.
8. Strongly disagree B. Somewhat disagree C. Uncertain D. Somewhat agree E. Strongly agree
9. It is important for patients to be involved in decisions about their own treatment and care.
10. Strongly disagree B. Somewhat disagree C. Uncertain D. Somewhat agree E. Strongly agree
11. It is important for patients to take primary responsibility for their own future health.
12. Strongly disagree B. Somewhat disagree C. Uncertain D. Somewhat agree E. Strongly agree

**Facilitation of Patient Involvement Scale**

1. My doctor provides me with all the information I need to make appropriate decisions.
2. Never B. Rarely C. Occasionally D. Sometimes E. Often F. Always
3. When choosing a treatment plan, my doctor ignores my opinions.
4. Never B. Rarely C. Occasionally D. Sometimes E. Often F. Always
5. When prescribing me a new medication, my doctor asks whether I have any questions about the drug and its possible side effects.
6. Never B. Rarely C. Occasionally D. Sometimes E. Often F. Always
7. My doctor discourages me from asking questions.
8. Never B. Rarely C. Occasionally D. Sometimes E. Often F. Always
9. My doctor explains all available treatment options so that I can make an informed decision.
10. Never B. Rarely C. Occasionally D. Sometimes E. Often F. Always
11. My doctor strongly encourages me to express any concerns I have about the current treatment.
12. Never B. Rarely C. Occasionally D. Sometimes E. Often F. Always
13. My doctor discourages me from expressing my views about my own condition.
14. Never B. Rarely C. Occasionally D. Sometimes E. Often F. Always
15. Healthcare providers make it difficult for me to participate in my own treatment and care.
16. Never B. Rarely C. Occasionally D. Sometimes E. Often F. Always

9. When I have doubts about treatment decisions, my doctor makes me feel it is difficult to communicate.

A. Never B. Rarely C. Occasionally D. Sometimes E. Often F. Always

**Family Adaption Partnership Growth Affection and Resolve Index**

1. When I encounter difficulties, I can get satisfactory help from my family.
2. Often true B. Sometimes true C. Rarely true
3. I am satisfied with the way my family discusses various matters with me and shares my problems.
4. Often true B. Sometimes true C. Rarely true
5. When I want to engage in new activities or personal development, my family is accepting and supportive.
6. Often true B. Sometimes true C. Rarely true
7. I am satisfied with the way my family shows concern and care for my emotions.
8. Often true B. Sometimes true C. Rarely true

5. I am satisfied with the way my family spends time with me.

A. Often true B. Sometimes true C. Rarely true

**Mac Arthur Scale of Subjective Social Status**

The following 10 numbers represent different levels of socioeconomic status. A score of 10 represents the most affluent people, with the highest income, highest level of education, and the most prestigious jobs; a score of 1 represents the poorest people, with the lowest income, lowest level of education, and the least prestigious jobs or no job at all.

1. Based on the level of socioeconomic development in your province, where do you think your current status stands compared with other people in your province?

1 2 3 4 5 6 7 8 9 10

2. Compared with the people around you (such as family members, colleagues, friends, classmates, neighbors, or community members), where do you think your current status stands?

1 2 3 4 5 6 7 8 9 10
